# Insights from designing an artificial cascade catalysis system using principles from substrate channeling in enzymes†

**DOI:** 10.1039/d5sc02781k

**Published:** 2025-06-30

**Authors:** Frances A. Houle, Peter Agbo, Junko Yano

**Affiliations:** a Chemical Sciences Division, Lawrence Berkeley National Laboratory Berkeley CA 94720 USA fahoule@lbl.gov; b Molecular Biophysics and Integrated Bioimaging Division, Lawrence Berkeley National Laboratory Berkeley CA 94720 USA

## Abstract

Generalizing the key requirements of highly-selective, multi-step chemical conversions involving spatially separated reaction centers remains one of the grand challenges of chemistry. Much work towards this effort has focused on decomposing multi-step conversions into their constituent reactions, whose intermediates are successively upgraded in a chemical cascade *via* diffusion from center to center. This approach for synthesizing more complex molecules takes its cues from biochemical networks, where near-unit conversion of even complex carbohydrates is achieved by upgrading chemical precursors *via* enzymatic cascades. In this computational study we examine a simple cascade involving coupled Ag and Cu catalysts that sequentially converts CO_2_ to CO and then CO_2_ and CO to reduced products, generically named CO_2_Product and COProduct. The system architecture is inspired by the phenomenon of biological substrate channeling, and components are examined to evaluate their effects on conversion efficiency in the cascade. Aside from a substrate channel linking two reaction centers, we find efficient cascades must also incorporate directional substrate diffusion, compartmentalization of the reaction centers, and proper timing of substrate arrival at the active center. We make explicit linkages between these requirements and chemical conversion in known biological systems, revealing additional control elements that could be incorporated.

## Abstract 2

A modeling study shows that catalytic cascades for artificial photosynthetic systems can be made highly efficient by incorporating multiple biological principles, including confinement, directional diffusion and timing modulation.

## Graphical abstract

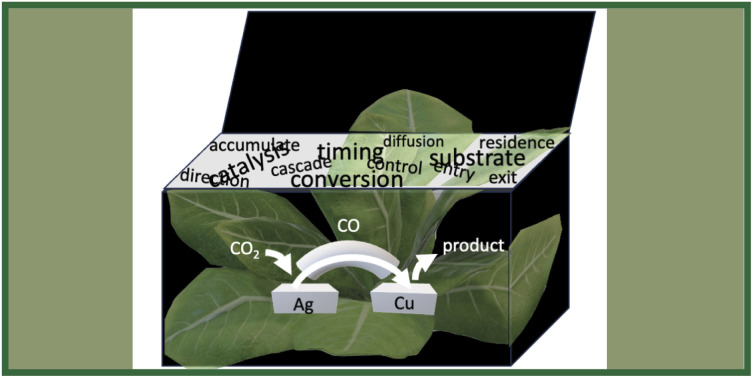

## 1 Introduction

In nature, complex reaction sequences involving multiple reaction centers are locally managed by structural elements within proteins that place reaction centers near each other and couple them using molecular assemblies that direct substrate motion between them. This process, called substrate channeling, confines the critical functions of the cascade to a well-defined region in space. The molecular assemblies, or channels, that provide coupling confine motion through their physical properties. For example, they can be relatively impermeable, tube-like structures that constrain diffusion to a region within them or contain ionic regions whose negative and positive charge promote hopping of a charged substrate along a well-defined path. Examination of the characteristics of substrate channeling systems has provided important understanding of how they might be incorporated into artificial systems,^1,2^ thereby improving the selectivity and efficiency of human-made catalytic cascades. In this work, we consider how channels might be used in a central artificial photosynthetic reaction, the (photo)electrochemical conversion of CO_2_ to reduced hydrocarbons and oxygenates. This reaction cannot be performed selectively with a single catalyst, according to what is known today,^3^ and extensive studies have been made of cascades where Au or Ag catalysts decompose CO_2_ to form CO which then is reduced by Cu as a route to improved efficiency and selectivity.

The experimental artificial cascade demonstrations performed so far have co-located the Ag (or Au) and Cu catalysts in a spatially well-defined arrangement under the assumption that proximity promotes a cascade,^4–6^ although it has been recognized that there are advantages to having the catalysts placed in separated electrolyzers.^7^ Electrolyte flow in these systems enables reactants to be supplied continuously and products to be accumulated downstream. At the micron-scale, flowing fluids are always accompanied by a stagnant boundary layer in which diffusion is the mechanism for delivery of reactants to and between two catalytic regions. Detailed simulations of a realistic yet simplified model for a catalytic cascade within the boundary layer have demonstrated that proximity alone is insufficient, however, because diffusion within it is driven by concentration gradients only.^8^ These gradients are not strongly directional because an intermediate desorbing from a catalytic surface can go anywhere. It is necessary to flood the boundary with the cascade's intermediates in order for the cascade to work when direct flow cannot occur.

Substrate channeling can potentially offer improvements. A cell is the governing model, where internal structures provide the necessary elements to organize chemical conversions across multiple time and length scales. These structures successfully control complex processes such as metabolism^9^ and permit combinations of catalytic reactions that require incompatible environments.^10^ Artificial chemo-enzymatic and organic synthetic cascades have been designed using several key design elements. The multiple catalysts involved in the cascade are close but spatially isolated from the other, and reactants move between the catalysts *via* diffusion in a channel rather than by random diffusion.^11,12^ Work on artificial multienzyme systems has shown a requirement that active sites more than 1 nm apart that require substrate diffusion between them to execute the chemical cascade be in some kind of a constraining structure to ensure efficient transfer.^1,2^ A channel can do this, and can couple reactions at distances to about 10 nm.^2^ Computational studies have investigated substrate channeling in natural systems, providing additional insights to important characteristics. One study looked at electrostatic channeling in an artificial metabolon, and identified leakage from the channel as a major cause of cascade inefficiency.^13,14^ Another study considered the physics of a 2-catalyst cascade.^15^ It examined spatial arrangement effects on the interplay of substrate consumption and inter-catalyst diffusive transport, and how the steric confinement of the substrate affects the interactions between catalysts and substrates.

Studies of natural systems show us that more is involved beyond spatial placements and the existence of channels, however. Transfer of indole in tryptophan synthase has revealed that simply providing a channel within the complex is not enough. Allosteric interactions are a key element of the function of this system, serving to guide and coordinate the chemical reactions in the cascade sequence.^16^ Such a mechanism could actively control the timing of the cascaded reactions, for example only passing an intermediate to its downstream reaction center when that center is ready to receive it: that is, is in the catalytic cycle's resting state. A recent investigation of a process involving a cascade of photoexcitation–redox reactions by a photoactive dye – molecular water oxidation catalyst diad showed that thermal reactions are rate determining at steady-state, stalling the catalytic sequence until they are completed.^17^ While the diad is in the thermal-only state, photoexcitations continue but are wasted. A similar consideration would apply to low, single-pass efficiencies for an artificial catalytic cascade reaction involving a flowing substrate: if the catalyst is not ready to react with the substrate when it is nearby, that opportunity is lost. A series of matched catalysts that operate in a cascade, where the product of one catalytic center is a feedstock for another catalytic center and arrives at the right time, offer the potential of improved selectivity and more efficient use of electrons to drive the reaction.^4–6^

What is the best way to use biological principles to design cascades for artificial CO_2_ reduction systems? Although active substrate convective flow is an effective strategy for driving a cascade,^11,12^ natural systems work well without it. Can diffusion through a substrate channel alone ensure a useful artificial cascade for CO_2_ reduction as is the case for metabolism?^9^ Is allostery specifically needed in artificial systems? What artificial factors are needed to coordinate and synchronize the function of two cascaded catalysts as seen in the tryptophan synthase system?^16^ In this work, we describe a computational study that seeks to answer these questions using 3-D stochastic chemical reaction-diffusion simulation of a highly simplified description of the Ag–Cu electrocatalytic CO_2_ reduction cascade, a useful model system.^8^ On Ag, CO_2_ is reduced to CO.^18^ Both CO and CO_2_ are reduced by Cu to a number of C_1_ and C_2_ products.^3^ Including both reaction channels for Cu provides a useful assessment of how well the cascade operates in various architectures when it is coupled by diffusion alone because it allows the competition between the background reaction with CO_2_ and the cascade reaction to be assessed. The Cu chemistry is limited to 2-electron products for the purposes of this work, which is unrealistic but does allow the details of how the whole reaction-diffusion system operates to be evaluated.

The results show that the conceptual framework that has been developed for natural systems – substrate channeling and allostery for directional movement and coordination between the catalytic centers – is incomplete when translated directly into an artificial system. Elements that are also required are a means of guiding the substrate into the catalytic assembly in the first place or signaling readiness between two catalysts when allosteric motion is absent. In this work we consider what types of control elements are needed and present an artificial design concept employing them that successfully drives an efficient catalytic cascade.

## 2 Methods

**Fig. 1:**
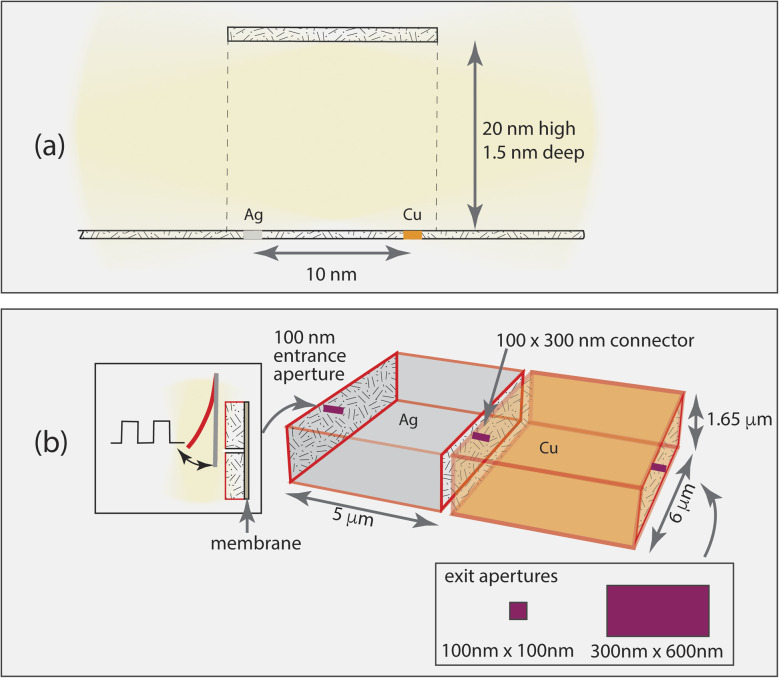
Outline of simulation volumes. (a) Single channel connecting two catalytic centers. The channel structure is 1.5 nm wide × 20 nm high × 9–11 nm long. 1.5 nm × 1.5 nm films of Ag and Cu are provided in one of three locations: each just outside the channel openings (separated by about 10 nm center-to-center), each just inside the channel openings with a ∼10 nm separation, and both near the center, separated by 1 nm. The Cu and Ag films are assumed to have 2 catalytic sites (∼10^14^ sites per cm^2^). The channel height is selected to enable each simulation sub-compartment to include at least one CO_2_ molecule. (b) Coupled catalytic compartments (both ∼5 × 6 μm) with connecting tube, gated inlet and exit openings. Insets show the placement of a cantilever for gating and the exit aperture locations and sizes. A permselective membrane that blocks exit of the CO intermediate is included. Catalyst films cover the interior walls of the compartments.

Two catalytic centers operating in an open environment are not efficiently coupled into a cascade.^8^ The calculations performed in this work evaluate a progressively more complex series of architectures coupled to functions to identify important elements to include in a cascaded system. Full details on model construction and the simulation methods are provided in the ESI Notes 1–4,† and are briefly summarized here. The simulations fall into two groups: evaluation of the characteristics of a simple, fully passive connecting tube (Fig. 1a), and evaluation of the characteristics of coupled compartment architectures that provide more control over the reactants (Fig. 1b). Both models involve the same chemical mechanisms as shown in Table S1,† electrocatalysis of CO_2_ by Ag and Cu at −1.4 V *vs.* SHE. The highly simplified cascade is production of CO on the first catalyst, Ag, and reaction of both CO and unreacted CO_2_ on the second catalyst, Cu to form COProduct and CO_2_Product which are generic names for the reduction products formed. Electrolyte reactions are neglected: the CO_2_ concentration in the system is assumed to be controlled by the diffusive flux through the inlet and reactions at the electrodes.

The calculations were performed using the general-purpose stochastic reaction-diffusion simulation package Kinetiscope.^19^ Its use for coupled reaction-diffusion in spatially distributed catalytic systems has been described previously.^20,21^ Simulations were performed for a total reaction time of 20 s for the simple channel model, and 10 s for the connected compartments model. The simulation outputs are a full set of concentrations as a function of space and time throughout the entire system, catalytic current densities as a function of position, and occurrences information for the various reaction and diffusion steps taking place.

## 3 Results

### 3.1 The simplest case: a connecting channel

We start with the simple channeling element identified in natural and bioinspired systems, a tube-like structure. This channel provides a means for an intermediate to be transported from one specific catalytic site to another, and does not have leaky walls. For this case, the catalysts are 1.5 nm × 1.5 nm and have two catalytic sites each. Without a channel, two catalytic centers only operate together when the system becomes flooded with the intermediate.^8^ To evaluate whether an impermeable channel alone is sufficient to control a cascade, we examine 3 cases: catalysts located outside either end of the connecting channel, just inside the open ends of the connecting channel, and at the center of the connecting channel. CO_2_ can enter the channel from either end.

**Fig. 2:**
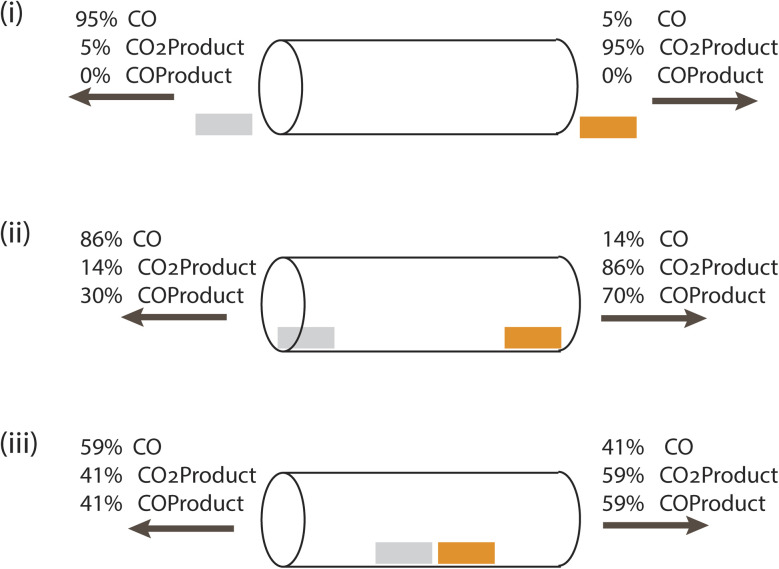
Schematic showing the performance of three single channel architectures, actual dimensions shown in Fig. 1(a). CO_2_ enters from both ends, and product gas mixtures produced by Cu (orange) and Ag (gray) catalysts exit from both ends. CO is only formed on Ag, COProduct and CO_2_Product are only formed on Cu. (i) Catalysts placed just outside the entrances of the tube (separated by 10 nm center-to-center), (ii) catalysts at the entrances of the tube with a 10 nm separation, and (iii) both catalysts near the center of the tube, separated by 1 nm.

Fig. 2 shows the partitioning of each product (totaling 100%) at the two ends of the channel for the three cases, and Table 1 shows the corresponding formation rate of each product. These streams show why the channel alone is insufficient. When the catalysts are external to it, diffusive motion of the cascade intermediate CO formed on Ag into the channel competes with its diffusion away from it, with only 5% crossing over to the Cu side. When CO reaches Cu, it can diffuse in any direction, and adsorption and reaction on Cu is uncompetitive. Placement of the catalysts just inside the channel openings improves cascading, with 14% of the CO formed reaching the Cu catalyst. The COProduct formation rate, however, is only 7 × 10^−4^% of the CO_2_Product formation rate. When the catalysts are 1 nm apart in the middle of the channel, far from the openings, the COProduct formation rate increases to 10^−2^%. However, loss of the intermediate out both ends of the channel remains the dominant process. This is a consequence of the undirected diffusion taking place in and around the channel. Use of a reaction-diffusion process to drive the cascade is not favored in this architecture.

**Table 1:** Steady state current densities and product formation rates on the Ag and Cu catalysts

Geometry	Ag current density, mA cm^−2^	Cu current density, mA cm^−2^	CO formation, Ag, mol s^−1^	CO_2_Product, Cu, mol s^−1^	COProduct, Cu, mol s^−1^
(i) Outside	−0.95	−0.95	6.66 × 10^4^	6.66 × 10^4^	0
(ii) At entrance	−0.95	−0.95	6.68 × 10^4^	6.69 × 10^4^	0.5
(iii) Near center	−0.95	−0.95	6.69 × 10^4^	6.7 × 10^4^	6.22

### 3.2 Structures that can improve on the simple channel

The simulations show that, as represented, a simple channel does not work to ensure efficient cascade catalysis. There is no mechanism to ensure that reactants diffuse in the desired direction or that the intermediate is efficiently captured by the second catalyst. In this section we will describe a set of simple elements that can be added to achieve controlled timing and encourage high single pass efficiency. The aim is to create designs that can be made and tested experimentally. Because large area fabrication of well-controlled nanometer-scale complex structures is highly challenging, designs that use molecular-scale architectures to control all aspects of the electrocatalysis are impractical. Instead, we move to the micrometer scale in order to facilitate tractable fabrication, enabling the concepts described here to be tested and improved *via* experimentation.

#### (A) Improvement # 1: one-way diffusive transit

The results in Fig. 2 show that allowing influx and egress from both ends of the channel introduces a significant loss pathway for the cascading intermediate. One of the functions of allostery in natural systems is to steer molecular motions in a desired direction. Artificial systems can incorporate this feature by using permselective membranes to ensure that some species are blocked but others allowed to pass. Here, placement of a membrane at one end, for example the entrance to the first cascade element, would enable the substrate CO_2_ to enter the system freely but block the intermediate CO from diffusing out of that opening. Commercial products that can perform this function are available. For example, the Polaris membrane is selective for CO_2_ permeation by a factor of 10–20 relative to CO, which would be effective in minimizing losses of the CO intermediate.^22,23^ Diffusion out of the exit aperture must similarly be one-way to prevent unwanted side reactions and for efficient product collection. This can be accomplished by ensuring that the concentrations of reactants and products are very low in the bulk electrolyte external to the compartment at that location, for example by using a flowing electrolyte. Because of the architecture used for the simple channel in the simulations, a direct test of this improvement using it was not made. Diffusive flux is proportional to surface area, and the catalyst areas are small (2.25 nm^2^) compared to the cross sectional area of the channel (30 nm^2^), required to reduce the computational cost of the simulations. The channel model is not well-suited to test this improvement, so its implementation has been combined with that of improvement # 2.

#### (B) Improvement # 2: compartmentalization

The literature on design of catalytic cascades ranging from synthetic biology engineering of metabolic processes^9^ to large scale synthesis^11^ emphasizes the need to provide structures that keep reactive regions close to one another yet chemically isolated, and provides a direct means of transferring intermediates from one reactive region to the next. Vesicles and liposomes are a natural construct to consider as they can be tuned in many ways to facilitate permeability control and stimulus response.^24^ Such compartments can be created in a side-by-side configuration and connected by narrow tubes that provide a transfer path between them but limit the diffusion rate of their contents from one compartment to the next because of the size of their cross sections. The advantages of this architecture have been demonstrated using a nanotube-vesicle network to not only control the location of a chemical reaction but also timing its migration between vesicles, using diffusion as the only transport mechanism.^25^ In those experiments, spherical vesicles in the range of 5–10 microns were connected using conduits 100–300 nm in diameter and 50–100 microns long. Over times of many minutes, the specific vesicle undergoing active chemistry changed abruptly, resulting in a sequential pulsing effect due to control over the reaction timing.

Taking the few microns size as a useful design point to facilitate movement of reactants, intermediates and products through the system, we can consider how to fabricate such a network for electrochemical cascades. An electrocatalytic cascade requires that the walls of the compartments be conducting, and be able to be coated with suitable catalysts. In a recent demonstration, 3D printing of an acrylate resin followed by pyrolysis to fabricate mesoscale systems of glassy carbon electrode compartments with specified architectures was reported.^26^ While the structures in that work were relatively large (∼100 micron scale), it is possible to 3D print structures with a few-micron scale using similar materials by 2-photon lithography.^27^ Addition of a membrane as described in Improvement # 1, and illustrated in Fig. 1b, ensures directional control (improvement # 1).

**Fig. 3:**
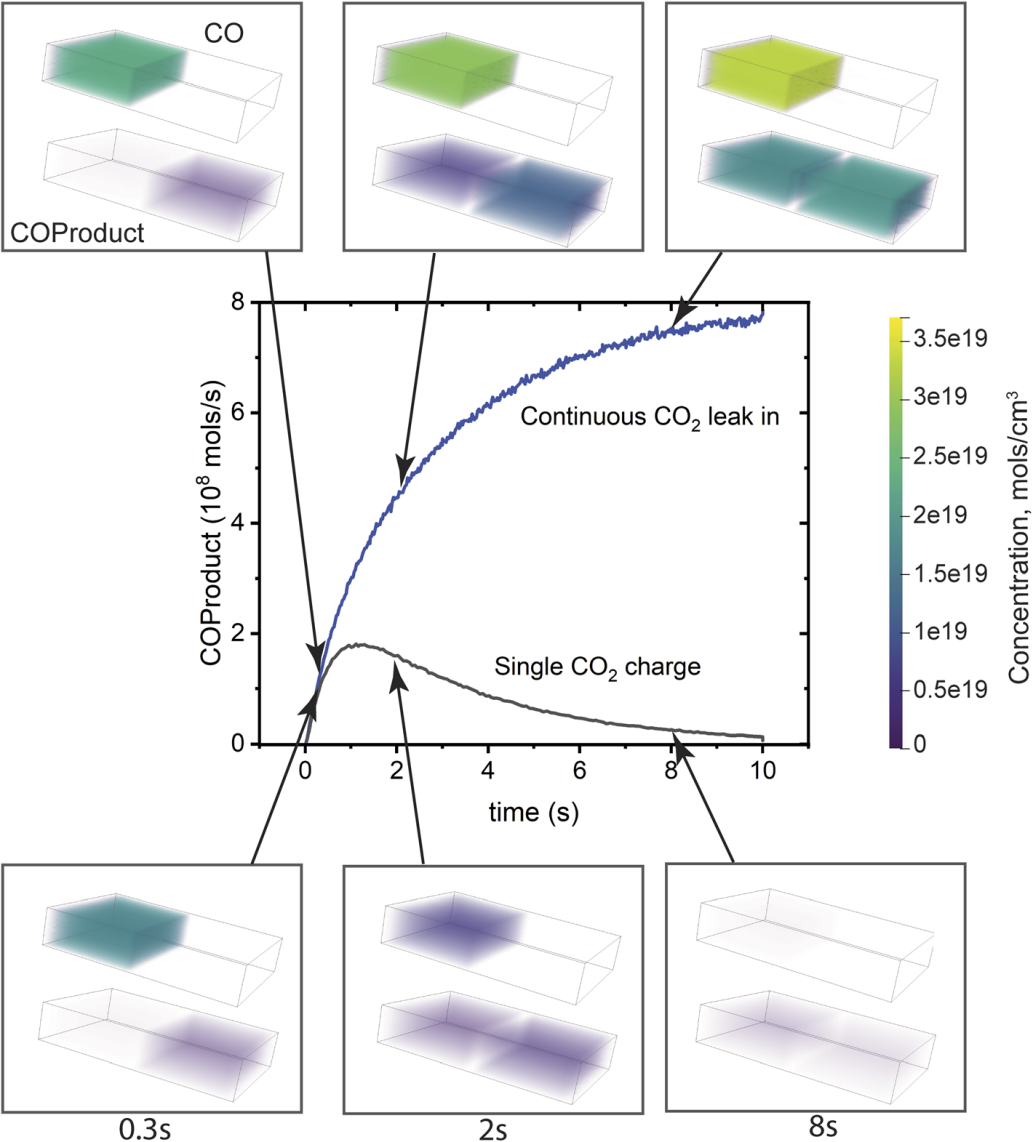
COProduct formation as a function of time in a coupled compartment cascade. The Ag compartment where CO is formed is on the left, and the Cu compartment where the product is formed is on the right. Two cases are compared: an initial single charge of the substrate CO_2_, and an initial charge with a continuous substrate leak into the system to replenish the charge. The plot shows the cascade product, COProduct, exit rate from the system (exit location is shown in Fig. 1b). It is at nearly steady state at 10 s, with a value near zero for the single charge and a constant for the continuous replenishment case. The contents of the two compartments are shown at times of 0.3 s, 2 s and 8 s for the two cases for both CO (upper image) and COProduct (lower image). COProduct can diffuse out of the system as well as back into the first compartment because there is no mechanism to inhibit backflow in this case. The color scale bar on the right indicates the concentrations of CO and COProduct.

To test whether provision of these elements alone are sufficient to have a cascade where sequential reactions are well-controlled, a simulation has been made assuming that the first compartment, which has the entrance to the system, is prefilled with the substrate CO_2_, mimicking the substrate injection process used in the vesicle experiments.^25^ Computationally, a many-micron-long conduit is impractical because diffusion through it is inefficient, so the conduit was shortened to 0.3 μm as described in ESI Note 3.† In the vesicle experiments, the pulsing behavior was attributed to building up a sufficiently large concentration of the intermediate in one vesicle so that the gradient driven diffusion into the adjacent vesicle would abruptly become very rapid, triggering the reaction there and rapidly consuming the intermediate. As can be seen in Fig. 3, this indeed occurs. However there is also some back-diffusion of the COProduct into the first chamber before the system completely empties out. If this type of system is to be run continuously, it is necessary for the first chamber to be refilled. If this is allowed by diffusion through the inlet, no pulsing occurs because the CO intermediate diffuses freely into the second chamber. It is evident that the connected vesicle-type system cannot control the timing of the chemistry if it operates continuously, as is desirable for a practical system.

#### (C) Improvement # 3: timing control

To control timing of a cascade, natural systems use allostery and signaling mechanisms such as accumulation of a critical concentration of reactants, arrival of electrons or protons, and light intensity to effect local changes in the microenvironment and start or stop specific chemical reactions.^28,29^ Artificial systems that control drug delivery by using photoexcitations to influence permeability have been described,^30^ but like the vesicle example studied here, are not designed to be refilled. In this study, we adapt the series of vesicles architecture to incorporate a gate-like structure to modulate the entry of the substrate into the compartment system, as illustrated in Fig. 1b. Two examples of such structures use a light signaling mechanism: an azobenzene-coated cantilever whose position can be controlled using UV light^31^ and photothermal deflection of a cantilever constructed from materials with dissimilar coefficients of thermal expansion.^32^ By modulation of the position of the cantilever, the substrate can enter the compartment system with a programmed frequency. In this work it is assumed that substrate concentration is constant in the reservoir external to the entrance, so there are no transport limitations on availability.

The data in Fig. 3 provide information on an appropriate modulation timing range for this system. The COProduct trace for the single CO_2_ charge case peaks at about 1 s, with a ∼1/*e* decrease by 4 s. This tells us that the conversion plus transit time is of the order of 1 s through the system. We selected a 2 s period (0.5 Hz) and a 4 s period (0.25 Hz) as the modulation times, assuming that 2 s would be the fastest time possible to clear the system, and 4 s would be a conservative estimate. Additional considerations are discussed in ESI Note 3.†

Using this architecture, three sets of run conditions are compared as described in Table 2, including a base case (0.5 Hz) and variations in exit aperture size and modulation frequency. The conditions are selected to assess their influence on the reaction efficiency and timing characteristics of the cascade. All cases assume the system has no CO_2_ in the compartments to start. A control simulation for the base case where both compartments are prefilled with CO_2_ (results not included here) showed that the process is dominated by conversion of the initially present substrate, eventually reaching a steady state like that of the initially empty compartment system.

**Table 2:** Characteristics of the three main dual-compartment system variations investigated

Case type	Modulation (Hz)	Exit aperture area (μm^2^)	Totals at exit (%)			
			CO_2_	CO	CO_2_Product	COProduct
Base	0.5, 3 cycles	0.01	1.2 × 10^−4^	0.15	0.089	99.8
Base + large exit	0.5, 3 cycles	0.18	2.1 × 10^−3^	2.02	0.082	97.8
Reduced modulation	0.25, 2 cycles	0.01	1.7 × 10^−4^	0.16	0.091	99.8

**Fig. 4:**
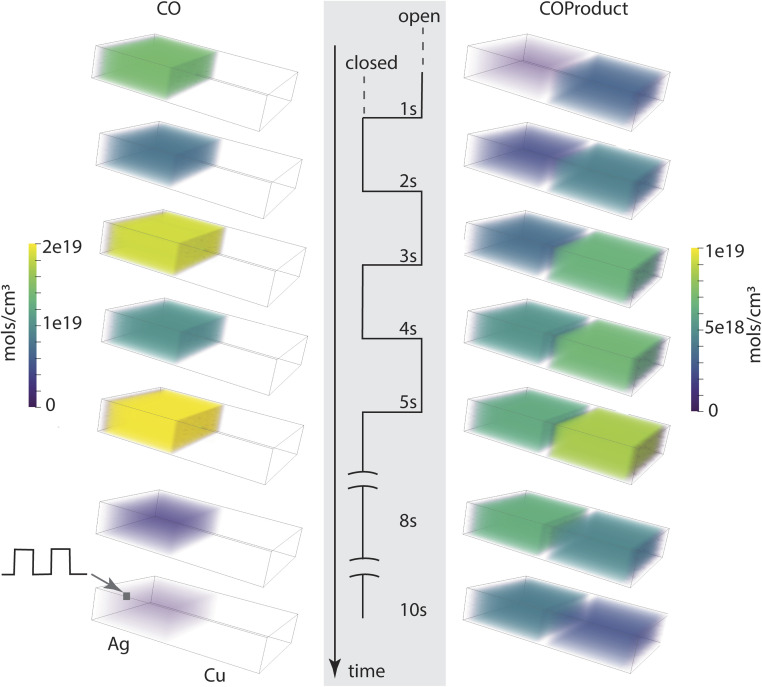
Compartment contents as a function of time point in the aperture open-close modulation cycle, for a total time of 10 s.

The fill-empty pattern of the two coupled compartments is shown for the base case in Fig. 4. The 3D images show that the contents of the two compartments are uniform within those volumes, indicating that diffusion within the volume is faster than diffusion between the compartments. The intermediate CO is only found in its source compartment (Ag catalyst) while the COProduct back-diffuses into the Ag compartment so is present in both and takes longer to clear out of the system. The most notable characteristic of the chemistry in this architecture is that CO and COProduct are the only significant components, indicating that the single pass conversion efficiency for CO_2_ to COProduct is very high. As shown in Table 2, all three cases have a conversion efficiency > 97%, the larger exit aperture system loses about 13 times more unreacted CO than the small aperture systems, while the aperture itself is 18 times larger. The simulations indicate that for the electrochemical kinetics used in this work, complete conversion in the Ag compartment is key to controlling the overall COProduct yield. This predicted single pass conversion efficiency is quite extraordinary for an electrochemical reaction, let alone an artificial cascade.

**Fig. 5:**
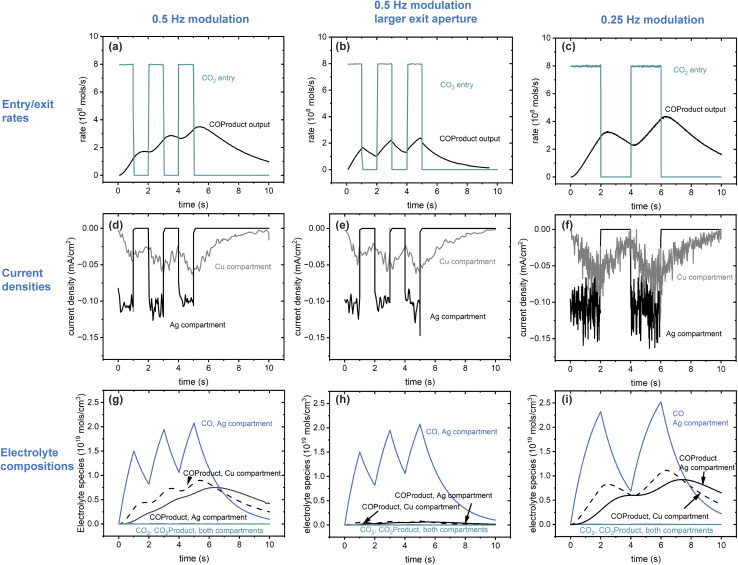
Line plots of key characteristics of the cascading system for the 3 cases in Table 2. (a)–(c) comparison of the CO_2_ entrance rate and COProduct exit rate, (d)–(f) comparison of the catalytic currents in the Ag and Cu compartments as calculated on a catalytic surface area of 0.05 μm^2^ at the same location in them, (g)–(i) comparison of the electrolyte compositions.

Line plots showing key characteristics of the three cases in Table 2 are shown in Fig. 5. The entrance aperture for the 3 cases is the same, 0.01 μm^2^, resulting in the same maximum CO_2_ entrance rate for the three (top three panels). This entrance rate is compared to the exit rate, which is the diffusion rate out of the exit aperture. Exit diffusion rates depend on concentration gradients as well as the exit surface area. In the three cases it is clear that the exit rate is smeared out compared to the entrance rate, reflecting the buffering effect that the architecture and the diffusion have on the transit of reactants and products through the system. Ideally the output modulation would match the input modulation with a geometry-dependent phase shift. The data for the large exit aperture and slower modulation cases come closest, likely because the residence time in the Cu compartment is much reduced when the aperture is large, and the slower modulation is better matched to the timing characteristics of the entire system. Although outside the scope of this project, these system response characteristics point to strategies that can be used to refine system dimensioning and operating parameters to ensure good temporal matching between input modulation and product exit waveforms. The data in the middle set of panels show that all 3 systems have the same electrochemical behavior. The bottom row of the figure shows that the electrolyte compositions are quite different, however. The CO concentration in the Ag compartment depends only on the modulation characteristics, while the reactant and product concentrations in the Cu compartment depends mainly on the exit aperture size.

## 4 Discussion

The outcomes of this study highlight which elements in natural systems are essential to their chemical functioning and need to be included in an integrated way into artificial systems, something not always revealed in mechanistic studies of the chemical reactions or of system architectures alone. We find that coupling two reactions through a substrate channel is insufficient for driving efficient cascade chemistries. Instead, effective substrate channeling is found to incorporate directional substrate fluxes (improvement # 1), and reaction compartmentalization (improvement # 2) and an ability to control reaction timing (improvement # 3), pointing towards combinations of key factors that must be considered in future efforts to rationally design artificial catalytic cascades. These features are necessary but likely not sufficient to achieve the selectivity of biochemical systems. In particular, allosteric regulation coupled to feedbacks also governs substrate turnover in many biological systems.

It is evident that simple placement within a biological structure does not ensure directionality without some additional constraint on motion. That directional substrate diffusion may assist efficient conversion in an artificial chemical cascade (improvement # 1) prompts further consideration of biological systems such as tryptophan synthase. In this system, directionality is achieved by mechanical means. Allosteric regulation of the protein is triggered by substrate egress into a 25 nm channel connecting the protein's α and β subunits.^16^ Conformational changes upon substrate capture by the protein reconfigures the system into a ‘closed’ state, prohibiting random walk of substrate out of the entry channel. This closed configuration results in stochastic location probabilities that amount to directional “channeling” of substrate molecules, and is effective in increasing the probability of substrate binding and reactivity at the enzyme active site. This principle has been recognized in biology, however its identification as being essential to artificial systems has not been investigated at the same level of detail as catalyst placement.^15^ Photosystem II also has a mechanism to open and close a proton gate, regulated by the hydrogen bonding network between water and amino acid residues.^33^ An artificial mechanical element used in the present simulations is a selectively permeable membrane, which controls directionality of CO diffusion by preventing its back-diffusion out of the first compartment. This is not an allosteric element, however, since its function is passive and determined by the membrane structure, and not controlled by signaling.

Another mechanism for ensuring directional motion the imposition of large concentration gradients, which govern diffusion rates. This is seen in the carbon concentrating mechanisms (CCMs) found in the carboxysome of many photosynthetic cell types. CCMs increase the ambient concentration of CO_2_ for downstream conversion to 1,5 ribulose bisphosphate by the enzyme Rubisco. This scheme is essential, as Rubisco's low binding affinity (high Michaelis constant, *K*_m_) for CO_2_ requires substrate concentrations that are higher than the ambient bulk concentration of CO_2_ (∼15 μM by Henry's law, calculated for 450 ppm CO_2_ in ambient air and a Henry's law constant of 3.4 M per atm in water).^34^ In this case, directional CO_2_ motion and concentration within the carboxysome occurs *via* active transport of cytoplasmic bicarbonate (HCO_3_^−^) anions across the organelle's membrane. Transport is facilitated by a concentration gradient that develops because of the action of the carbonic anhydrase enzymes inside the carboxysome, which catalyze the reversible dehydration of bicarbonate to CO_2_ and deplete its concentration relative to that in the cytoplasm. CO_2_ is unable to passively permeate the CCM membrane at appreciable rates and is not actively transported by bicarbonate transporters. The result is a directional motion and net buildup of CO_2_ inside the CCM, facilitating Rubisco's incorporation of this substrate into a sugar. Active transporters have not been incorporated into artificial systems, but may be elements to consider.

In both cases of directional motion in natural systems – governed by allostery or by active transport – concentration differences across a control point are key to ensuring this process occurs. Concentration gradients alone are not enough however, local structures that ensure efficient entry of the substrate into the control point must also be present.

While biological compartmentalization is often invoked at the level of organelles, the simple channel simulations reported in this study also demonstrate the importance of compartmentalization at the atomic level: without it reaction efficiency and selectivity for COProduct is very low because there is no mechanism to control the residence time around the catalytic center. This suggests that the relevance of compartments to cascaded chemical conversions is not limited to the micron scale (improvement # 2). A strong argument for the logic of atomic-scale compartmentalization is evidenced through the construction of proteins themselves, and comparing their activities with active-site only catalysts. The structural bulk of proteins is hard to justify until considering the role that such bulk plays in shielding reactive active-site intermediates and transition states from unwanted reactions with molecules in solution. These bulky catalytic systems also slow down migration of substrates and intermediates, and prevent cross-talk between the active sites. Crosstalk may occur in a variety of inorganic systems such as inorganic/organometallic catalysts, where d–d interactions between transition metal centers are well-documented.^35^ Such chemical interference is precluded by the organic insulation formed by polypeptides, speaking to the importance of compartmentalization for achieving high catalytic specificity. The inability for reactive centers to cross-react allows biochemical networks – where substrates are sequentially upgraded in an enzyme cascade – to function with almost unit selectivity. This general concept is now ported into the 2-compartment system disclosed here, through use of apertures that have dimensions much smaller than the bridged reaction volumes.

Timing the arrival of substrate to active centers (improvement # 3) represents a powerful handle for rate-matching the sequenced chemistries of a cascade to achieve highly-selective conversion. In the present study, such rate-matched reactions can be achieved through modulation of substrate influx. Modulation makes time for the surface reactions in the first compartment to go to completion before their products move into the second compartment. There are tradeoffs however, protracted intermediate residence and accumulation in the first compartment can reduce throughput/turnover frequencies of the overall cascade.

While timing the on/off states for catalytic cycling may be achieved through modulating substrate flow, timing turnover at active sites should, in principle, be possible to modulate by controlling the arrival of any component needed for the reaction. As a result, it seems likely that similar effects could be realized by modulating charge flow to metal active sites. This extends possible modulation approaches to include transient potentiostatic methods, where application of pulsed potentials at frequencies that enable synchronization between the two compartments permits the activation or deactivation of active site turnovers at will.

The operation of coupled intra-protein charge and substrate relays provides a useful example of how appropriately timing the arrival of substrate or charge at an active center ensures efficiency. One system is considered here as an example. Metal enzymes of the formate dehydrogenase family are comprised of Fe–S clusters that act as initial points of reduction/oxidation and convey charge to/from a molybdenum, tungsten or NADPH-dependent catalytic site that drives the reversible conversion of CO_2_ and formate. In *Desulfovibrio vulgaris* formate dehydrogenase (FdhAB), substrate turnover rates high as 1310 s^−1^ for formate oxidation and 315 s^−1^ for CO_2_ reduction to formate (two electron equivalents per reaction, 2620e^−^ per s and 630e^−^ per s respectively), have been reported.^36^ However, these rates are far slower than the intraprotein electron transfers (ET) between the Fe–S and the catalytic site, estimated to be in the range 10^6^ to 10^7^ s^−1^ using a semiclassical Marcus electron transfer model, commonly used for biological ET calculations (ESI Note 5†). The fast reaction rates indicate that intramolecular ET between FdhAB Mo/W active sites and the Fe–S clusters in this protein occur in multi-step tunneling transfers, with rate coefficients generally much greater than for actual substrate turnover. The result is a system in which the timescales for charge transfer and substrate turnover are so disparate that the intra-protein charge propagation is actually best characterized as a transient with short pulse times (the tunneling rate) and a pulse frequency governed by the chemistry, *i.e.* the frequency of oxidation/reduction events at the Fe–S cluster by exogenous donors. The evolution of this pulsed relay has resulted in a class of proteins that convert CO_2_ to formate with virtually 100% selectivity because electrons are always available for the reaction.

Consideration of these natural systems points to additional mechanical, active and time-dependent processes that might be included to control artificial catalytic cascades. Future efforts to refine artificial multi-step conversions may eventually require reproducing the physics of feedback-driven allosteric control to achieve conversion efficiencies and control on par with biochemical networks. However, it is important to note that the cascaded catalytic reactions in the simulations described here involve only two-electron processes, and the scope of processes that need to be part of a successful design may be much broader still. For example, the stoichiometry of higher-order (4e^−^ or greater) reductions will impose demands on cascade construction that are distinct from those investigated here and may make conditions such as the necessary substrate buffering in the first compartment, harder to satisfy. Inclusion of full electrolyte chemistry, which must be done for accuracy, is complex in compartments of this size (ESI Note 1†) because they are too small for pH to be defined, and the composition of the electrolyte will be dominated by fluctuations.^37^ Nevertheless, it is clear that the three major considerations distinguished in this work play key roles in cascade function and should inform future design efforts of cascade reactions in general. Extension of these design principles to gas-diffusion electrodes can be envisioned, with adaptations to enable reactant modulation and include sufficient water vapor.

## 5 Conclusions

In this work we describe the results of a computational study that reveal key functional components necessary for a catalytic cascade to be efficient and selective. The chemistry examined is a highly simplified version of CO_2_ reduction on Ag to form CO, and subsequent CO reduction on Cu (in competition with CO_2_ reduction on Cu) to form products. Studies of natural systems have pointed to the importance of substrate channeling to control chemical cascades involving enzymes. Simply providing a tube-like structure for substrate channeling between the two catalysts in an artificial system does not ensure that the cascade is efficient or results in the desired chemistry. High single-pass catalytic conversion efficiency is enabled by (i) controlling the directionality of motion from one catalyst to the next, (ii) providing modulation to coordinate reactions through timing, (iii) confining the catalytic regions in compartments that buffer the concentrations of reactants and intermediates by having small entrance and exit apertures and tailoring their sizes to control residence times in each region. When considered alongside examples of how biological systems work, these conditions can be seen to be necessary but probably not sufficient to ensure selectivity and efficiency in more complex chemical systems such as formation of multicarbon products from CO_2_. Nevertheless, the simulation results point to improvements that can be made to artificial cascade catalysis systems through new architectural designs.

## Author contributions

All authors have contributed to the investigation described in this manuscript, and have participated in draft writing, review and editing. All authors have given approval to the final version of the manuscript.

## Conflicts of interest

There are no conflicts of interest to declare.

## Supplementary Material

SC-OLF-D5SC02781K-s001

## Data Availability

The data supporting this study are available within the manuscript, in the associated ESI,† and from Zenodo at DOI: https://doi.org/10.5281/zenodo.14954706.

## References

[cit1] Lin J. L., Palomec L., Wheeldon I. (2014). Design and Analysis of Enhanced Catalysis in Scaffolded Multienzyme Cascade Reactions. ACS Catal..

[cit2] Wheeldon I., Minteer S. D., Banta S., Barton S. C., Atanassov P., Sigman M. (2016). Substrate channelling as an approach to cascade reactions. Nat. Chem..

[cit3] Nitopi S., Bertheussen E., Scott S. B., Liu X. Y., Engstfeld A. K., Horch S., Seger B., Stephens I. E. L., Chan K., Hahn C., Norskov J. K., Jaramillo T. F., Chorkendorff I. (2019). Progress and Perspectives of Electrochemical CO2 Reduction on Copper in Aqueous Electrolyte. Chem. Rev..

[cit4] Chan T., Kong C. J., King A. J., Babbe F., Prabhakar R. R., Kubiak C. P., Ager J. W. (2024). Role of Mass Transport in Electrochemical CO Reduction to Methanol Using Immobilized Cobalt Phthalocyanine. ACS Appl. Energy Mater..

[cit5] Gurudayal D. P., Malani S., Lum Y., Haussener S., Ager J. W. (2019). Sequential Cascade Electrocatalytic Conversion of Carbon Dioxide to C-C Coupled Products. ACS Appl. Energy Mater..

[cit6] Lum Y., Ager J. W. (2018). Sequential catalysis controls selectivity in electrochemical CO2 reduction on Cu. Energy Environ. Sci..

[cit7] Theaker N., Strain J. M., Kumar B., Brian J. P., Kumari S., Spurgeon J. M. (2018). Heterogeneously catalyzed two-step cascade electrochemical reduction of CO to ethanol. Electrochim. Acta.

[cit8] Houle F. A., Yano J., Ager J. W. (2023). Hurry Up and Wait: Managing the Inherent Mismatches in Time Scales in Natural and Artificial Photosynthetic Systems. ACS Catal..

[cit9] Agapakis C. M., Boyle P. M., Silver P. A. (2012). Natural strategies for the spatial optimization of metabolism in synthetic biology. Nat. Chem. Biol..

[cit10] Özgen F. F., Runda M. E., Schmidt S. (2021). Photo-biocatalytic Cascades: Combining Chemical and Enzymatic Transformations Fueled by Light. Chembiochem.

[cit11] Guo X. M., Xue N., Zhang M., Ettelaie R., Yang H. Q. (2022). A supraparticle-based biomimetic cascade catalyst for continuous flow reaction. Nat. Commun..

[cit12] Zhang M., Ettelaie R., Dong L. L., Li X. L., Li T., Zhang X. M., Binks B. P., Yang H. Q. (2022). Pickering emulsion droplet-based biomimetic microreactors for continuous flow cascade reactions. Nat. Commun..

[cit13] Liu Y. C., Hickey D. P., Guo J. Y., Earl E., Abdellaoui S., Milton R. D., Sigman M. S., Minteer S. D., Barton S. C. (2017). Substrate Channeling in an Artificial Metabolon: A Molecular Dynamics Blueprint for an Experimental Peptide Bridge. ACS Catal..

[cit14] Liu Y. C., Matanovic I., Hickey D. P., Minteer S. D., Atanassov P., Barton S. C. (2018). Cascade Kinetics of an Artificial Metabolon by Molecular Dynamics and Kinetic Monte Carlo. ACS Catal..

[cit15] Hinzpeter F., Tostevin F., Buchner A., Gerland U. (2022). Trade-offs and design principles in the spatial organization of catalytic particles. Nat. Phys..

[cit16] Dunn M. F., Niks D., Ngo H., Barends T. R. M., Schlichting I. (2008). Tryptophan synthase: the workings of a channeling nanomachine. Trends Biochem. Sci..

[cit17] Massad R. N., Cheshire T. P., Fan C. Q., Houle F. A. (2023). Water oxidation by a dye-catalyst diad in natural sunlight: timing and coordination of excitations and reactions across timescales of picoseconds to hours. Chem. Sci..

[cit18] Hatsukade T., Kuhl K. P., Cave E. R., Abram D. N., Jaramillo T. F. (2014). Insights into the electrocatalytic reduction of CO on metallic silver surfaces. Phys. Chem. Chem. Phys..

[cit19] Hinsberg W. D. and Houle F. A., Kinetiscope, accessed Oct 1, 2024, https://www.hinsberg.net/kinetiscope

[cit20] Houle F. A. (2019). Reaction-Transport Coupling in a Nanostructured Porous Electrode. J. Phys. Chem. C.

[cit21] Houle F. A. (2021). Adaptive response by an electrolyte: resilience to electron losses in a dye-sensitized porous photoanode. Chem. Sci..

[cit22] Korelskiy D., Grahn M., Ye P., Zhou M., Hedlund J. (2016). A study of CO2/CO separation by sub-micron-oriented MFI membranes. RSC Adv..

[cit23] Lin H. Q., He Z. J., Sun Z., Vu J. M., Ng A., Mohammed M., Kniep J., Merkel T. C., Wu T., Lambrecht R. C. (2014). CO2-selective membranes for hydrogen production and CO2 capture - part I: membrane development. J. Membr. Sci..

[cit24] Velasco-Garcia L., Casadevall C. (2023). Bioinspired photocatalytic systems towards compartmentalized artificial photosynthesis. Commun. Chem..

[cit25] Sott K., Lobovkina T., Lizana L., Tokarz M., Bauer B., Konkoli Z., Orwar O. (2006). Controlling enzymatic reactions by geometry in a biomimetic nanoscale network. Nano Lett..

[cit26] Narita K., Citrin M. A., Yang H., Xia X. X., Greer J. R. (2021). 3D Architected Carbon Electrodes for Energy Storage. Adv. Energy Mater..

[cit27] Saha S. K., Wang D., Nguyen V. H., Chang Y. N., Oakdale J. S., Chen S. C. (2019). Scalable submicrometer additive manufacturing. Science.

[cit28] Jain A., Zoncu R. (2022). Organelle transporters and inter-organelle communication as drivers of metabolic regulation and cellular homeostasis. Mol Metab.

[cit29] Kong S. G., Wada M. (2016). Molecular basis of chloroplast photorelocation movement. J. Plant Res..

[cit30] Fomina N., Sankaranarayanan J., Almutairi A. (2012). Photochemical mechanisms of light-triggered release from nanocarriers. Adv. Drug Deliv. Rev..

[cit31] Ji H. F., Feng Y., Xu X. H., Purushotham V., Thundat T., Brown G. M. (2004). Photon-driven nanomechanical cyclic motion. Chem. Commun..

[cit32] Li Z. W., Ye Z. Y., Han L. L., Fan Q. S., Wu C. L., Ding D., Xin H. L. L., Myung N. V., Yin Y. D. (2021). Polarization-Modulated Multidirectional Photothermal Actuators. Adv. Mater..

[cit33] Hussein R., Ibrahim M., Bhowmick A., Simon P. S., Chatterjee R., Lassalle L., Doyle M., Bogacz I., Kim I. S., Cheah M. H., Gul S., de Lichtenberg C., Chernev P., Pham C. C., Young I. D., Carbajo S., Fuller F. D., Alonso-Mori R., Batyuk A., Sutherlin K. D., Brewster A. S., Bolotovsky R., Mendez D., Holton J. M., Moriarty N. W., Adams P. D., Bergmann U., Sauter N. K., Dobbek H., Messinger J., Zouni A., Kern J., Yachandra V. K., Yano J. (2021). Structural dynamics in the water and proton channels of photosystem II during the S to S transition. Nat. Commun..

[cit34] Sander R. , Henry's Law data, Carbon Dioxide, accessed February 25, 2025, https://webbook.nist.gov/cgi/cbook.cgi?ID=C124389&Mask=10#Solubility

[cit35] Powers I. G., Uyeda C. (2017). Metal-Metal Bonds in Catalysis. ACS Catal..

[cit36] Oliveira A. R., Mota C., Mourato C., Domingos R. M., Santos M. F. A., Gesto D., Guigliarelli B., Santos-Silva T., Romao M. J., Pereira I. A. C. (2020). Toward the Mechanistic Understanding of Enzymatic CO2 Reduction. ACS Catal..

[cit37] Li S. R., Kwon S., Goddard W. A. G., Houle F. A. (2023). A stochastic description of pH within nanoscopic water pools. Cell Rep. Phys. Sci..

