# Supplementary material for: Insights from designing an artificial cascade catalysis system using principles from substrate channeling in enzymes
Source: Chem Sci. 2025 Jun 30;16(30):13667–77. doi: 10.1039/d5sc02781k (PMC12231582; doi:10.1039/d5sc02781k)
Supplement: SC-016-D5SC02781K-s001 [file SC-016-D5SC02781K-s001.pdf]

Supplementary Information (SI) for Chemical Science.
This journal is © The Royal Society of Chemistry 2025

Electronic Supplementary Information

Insights from designing an artificial cascade catalysis system using principles from substrate channeling in enzymes

Frances A. Houle[a,b] * Peter Agbo,[a,b] and Junko Yano[b]

[a] Chemical Sciences Division, Lawrence Berkeley National Laboratory, Berkeley, CA 94720
[b] Molecular Biophysics and Integrated Bioimaging Division, Lawrence Berkeley National Laboratory, Berkeley, CA 94720

* Corresponding author: fahoule@lbl.gov

Table of contents:

Supplementary Note 1: Chemical reactions and molecular diffusion

Supplementary Note 2: The simple channel architecture

Supplementary Note 3: The coupled compartments architecture

Supplementary Note 4: Simulation techniques

Supplementary Note 5: Comparative timescales of intramolecular electron transfer (ET) vs substrate turnover in *Desulfovibrio vulgaris* formate dehydrogenase

Supplementary Note 1: Chemical reactions and molecular diffusion

The catalytic reaction scheme used in this work for both the simple channel model and the coupled compartments model is based on one reported in a previous publication that looked at the role of proximity in catalytic cascades in an open (unconstrained) system.[1] In that work, only one reaction could occur on each catalyst, i.e. reaction steps (1) and (3) below. To assess reaction efficiency, the competing CO_2 reaction step (2) is added in the present work.

The overall chemistry is

On Ag: $CO_2 \rightarrow CO$ (1)

On Cu: $CO_2 \rightarrow CO_2\text{Product}$ (2)

$CO \rightarrow \text{COProduct}$ (3)

where CO_2Product and COProduct are 2-electron reduction products. It is assumed that no chemical reactions occur in the aqueous electrolyte and that OH^- diffuses out of the compartments fast enough that local pH does not change. Modeling of local pH in such small volumes is very complex.[2] As shown in that study, pH is not defined for water pools less than 3.3μm in radius at pH 10 (in the range found for steady state CO_2 reduction by Cu)[3] because water ions are not present continuously, but fluctuate due to the kinetics. This radius corresponds to a minimum volume of 150μm^3. The volume of one catalytic compartment in the present work is 49.5μm^3, well below the minimum. Accordingly, the CO_2 — HCO_3^- — CO_3^{2-} coupled equilibria known from bulk measurements may look very different in these pools even if the rate coefficients are independent of pool size because intermittent ion presence will cause large concentration fluctuations. Extensions of the models reported here to include full electrolyte chemistry will need to incorporate a stochastic rather than a continuum description in order to take these fluctuations into account and quantify the importance of HCO_3^- and CO_3^{2-} formation within the compartments.

CO, CO_2, COProduct and CO_2Product diffuse into, out of, and within the system using Fickian kinetics. All diffusion coefficients are set to 2 x 10^{-5} cm^2/s, a value close to those for CO, CO_2 and formate in water.[4-6] Adsorption of CO and CO_2 onto the catalyst is treated as a net diffusion process, with a sticking probability of 5x10^{-2} which results in a current density for CO_2 reduction on Ag in the range of 1 mA/cm^2.[1]

Details of the reaction stoichiometries and their Butler-Volmer rate coefficient parameters are presented in **Table S1**. As noted in the Table, to support deeper analysis of the simulation results, marker species are built into the reaction mechanism. The markers play no role in the chemistry or the reaction rate calculations, they only serve to count the number of times a particular reaction step occurred in that location over the simulated time period. This useful technique is used routinely in our simulation studies, a more extensive example relevant to photocatalysis is found in Ref [7].

Table S1. Simplified CO_2 and CO reduction reaction schemes used in this work, rate coefficients from CO_2 reduction on Ag chemistry[8]

Ag reactions	Cu reactions	Formal potential (V vs SHE)	a	Standard rate constant (cm s^{-1})	Notes
$CO_2 + * \rightarrow *CO_2$	$CO_2 + * \rightarrow *CO_2$	--	--	--	(a), (b)
$*CO_2 + e^- + H_2O \Leftrightarrow$ Intermediate	$*CO_2 + e^- + H_2O \Leftrightarrow$ Intermediate	0.03	0.5	0.241	
Intermediate + e- $\rightarrow$ OH^- + CO	Intermediate + e- $\rightarrow$ OH^- + CO_2Product	-0.35	0.5	2.16 x 10^{-7}	(c)
	CO + * $\rightarrow$ *CO				(a)
	$*CO + e^- + H_2O \Leftrightarrow$ Intermediate	0.03	0.5	0.241	
	Intermediate + e- $\rightarrow$ OH^- + COProduct	-0.35	0.5	2.16 x 10^{-7}	(d)

a. Net adsorption is assumed to be controlled by diffusion to the surface (2x10^{-5} cm^2/s) and a sticking probability of 0.005, with a net adsorptive diffusion coefficient of 10^{-7} cm^2/s.
b. * = active site
c. Marker for this step is CO_2Product_formed, used to count total product formation rates as a function of position
d. Marker for this step is COProduct_formed, used to count total product formation rates as a function of position

Supplementary Note 2: The simple channel architecture

In the present study, the simple channel is a structure with a rectangular cross section of 1.5 nm wide x 20 nm high, 9-11 nm long (**Figure 1a in the main paper**), placed in liquid electrolyte. 1.5 nm x 1.5 nm films of Ag and Cu are provided in one of three locations separated by a distance measured between the closest edges of the films: each just outside the openings of the channel (separated by 9 nm), each just inside the openings of the channel with a 9 nm separation, and both near the center of the channel, separated by 1 nm. Center to center distances are 10.5, 10.5 and 2.5 nm, respectively. A 2.25 nm^2 film is assumed to have 2 catalytic sites (~10^{14} sites/cm^2). The channel is divided into a set of 1 nm wide compartments. Their 20 nm height is necessary for computational efficiency reasons, ensuring that each of the compartments contains 1 dissolved CO_2 at the overall 30 mM concentration expected for CO_2 in water at the beginning of the simulation. External to the channel is a reservoir of electrolyte saturated with CO_2, stirred sufficiently rapidly that products are promptly swept away after they are formed and the CO_2 concentration remains constant. CO_2 in solution diffuses randomly in all directions, and when it enters the channel from either end it can simply diffuse through or interact with one of the catalysts. **Figure S1** is an outline of the simulated architecture, showing the 3D layout to scale.

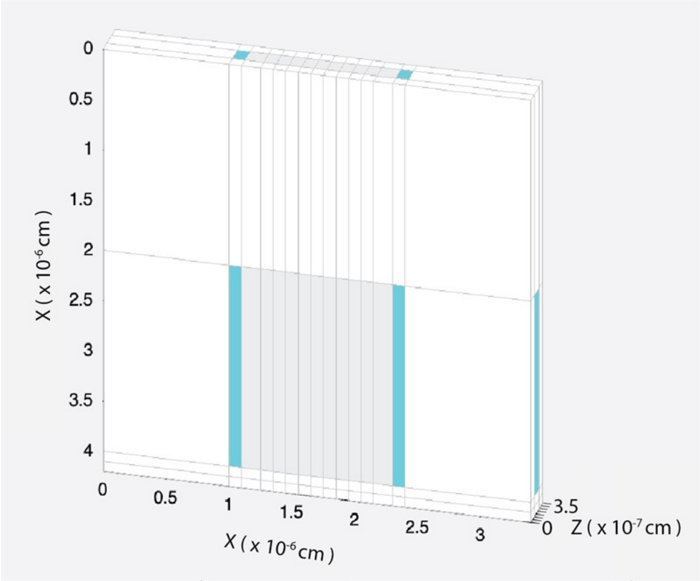

Figure S1. Outline of the 3-layer (front – to - back) multi-compartment structure used in this work. The channel itself is located in the middle layer. Turquoise compartments are filled with electrolyte which is assumed to be rapidly stirred and thus of constant composition. White and gray compartments are empty and for structural purposes only. The schematic is for the case where the catalysts are placed just inside the entrances to the channel. They have a 10 nm separation. The other cases have slightly different separations.

Supplementary Note 3: The coupled compartments architecture

This overall architecture is more similar to intracellular structures, where distinct reactive regions are well-defined. A key design principle for this architecture is that it can be fabricated as an artificial system using literature methods, so it is at the micron scale rather than the nanoscale as captured in the simple channel model. The aim of this architecture is to control both timing of the catalytic reactions and ensure a highly efficient cascade. The system consists of two compartments as shown in **Figure 1b** in the main paper and **Figure S2**, with liquid electrolyte on the entrance side, with either gas or liquid on the exit side. Each compartment has internal dimensions of 5.996 x 4 x 1.646 microns. The compartments are connected by a tube 100 nm x 100 nm internal cross section and 300 nm long. The compartment-tube-compartment system has a 100 x 100 nm entrance aperture and an exit aperture of variable size as shown in Figure 1b and described in **Table 2** in the main paper. At the entrance there is a CO_2 reservoir as included in the simple tube model that makes available a constant source of CO_2 via diffusion through the aperture. A cantilever to modulate diffusion through the entrance aperture is located as shown in Figure 1b. It should be noted that in Kinetiscope this cantilever does not need to be included explicitly: there is a capability to modulate diffusion through the entrance virtually by dynamically altering the kinetics at the desired frequency. A permselective membrane is located just inside the first compartment, covering the entrance aperture. This membrane enables CO_2 to enter but prevents CO from back-diffusing. At the exit, all evolved products are assumed to be swept away rapidly, and there is little CO_2 so none can enter the coupled compartment system from there. This arrangement is similar to that for a gas diffusion electrode where CO_2 is only available to the catalyst from one side of the system. The 4 sidewalls of each compartment are the electrode. Catalyst films cover the interior of these walls, and are biased at -1.4V vs SHE to drive electrochemical reactions involving the species dissolved in the internal electrolyte. The electrochemical reactions take place in a 1 nm thick interfacial electrolyte – electrode region. The catalytic site concentration is 10^{14} cm^{-2} as was used in the tube model, and the external CO_2 concentration is also 30 mM. **Figure S2** shows the outline of the simulated architecture with the

3D layout of sub-compartments to scale. As for the channel model, the sub-compartments are sized according to their function for computational efficiency.

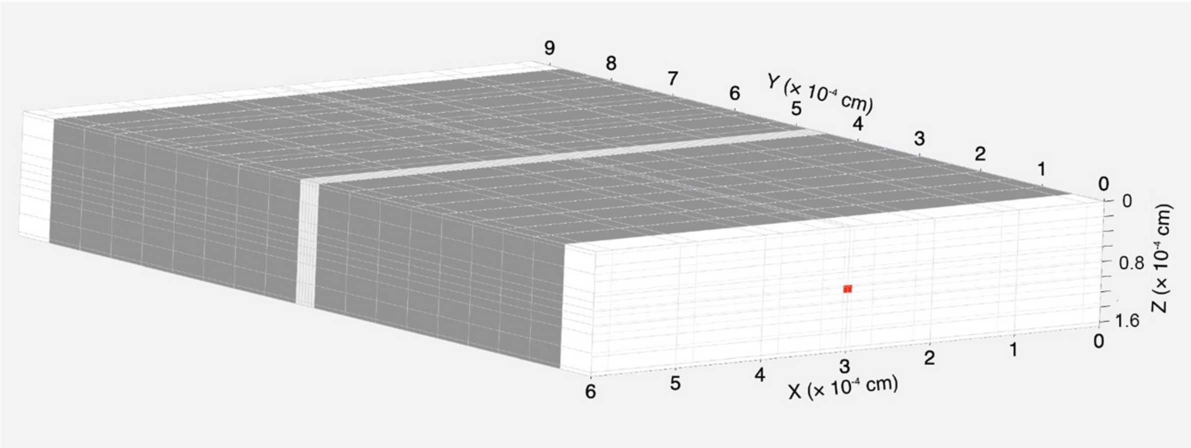

Figure S2. Outline of the 2-compartment model investigated in this work, showing the layout of compartments. The dark gray areas are electrodes covered with catalyst on the interior, the light gray area is a separator with the connecting channel at its center, and the white areas are structural elements. The small red rectangle in the middle of the white end section is the exit channel. The compartments are built from many sub-compartments, whose dimensions are selected to enable accurate diffusion rates in competition with reactions on the catalyst surfaces.

The simulation results provide some information on the native time constant associated with filling, diffusion and reaction, and emptying in the 2-compartment system. As described in the main paper and shown in Figure 3, a phenomenological assessment of the native time constant is available from the simulations. The characteristics of the system can be considered directly to provide an estimate. The base case involves 3 open-and-close cycles for the gate, with a frequency of 0.5 Hz. In one second, which is ½ of a 0.5 Hz period, a species with a $2x10^{-5}$ cm^2/s diffusion coefficient will traverse a distance of about 45 μm. This is about 4 times the total length of the 2-compartment system of 10 μm, which might indicate that 0.25 s would be enough to move through the system assuming concentration gradients are constant. However, there is a significant constriction in the middle that will slow down the overall diffusion rate from end to end. This is because the diffusion rate in units of concentration/time between two points depends not only on the diffusion coefficient and the concentration gradient of the diffuser, but also on the cross sectional area of the diffusion path. This area is 0.01 μm^2, much smaller than the compartment cross section of 6.58 μm^2. Additionally, the concentration gradients change continuously during the gating process. As shown in **Table S1** and **Figure 4** of the main paper, a period of 0.5 Hz is sufficient to transfer nearly all CO intermediate species from the first compartment into the second one. This indicates that the natural entrance – to -exit time constant for the system considered here is much longer than 2 Hz and probably comparable to 0.25 Hz.

Supplementary Note 4: Simulation techniques

The calculations reported in this work were performed using the general-purpose stochastic reaction-diffusion simulation package Kinetiscope.[9] Its use for coupled reaction-diffusion in

spatially distributed catalytic systems has been described previously.[10, 11] The computational method[12, 13] is a type of kinetic Monte Carlo simulation that performs a random walk across event space. It provides a rigorously accurate solution to the master equation for the system, and because it does not use coupled differential equation integration, it is useful for spanning broad ranges of reaction rates, spatial dimensions and time, and supports simulations of 3-dimensional coupled reaction-diffusion systems.[14] If an accurate reaction mechanism and rate and diffusion coefficients are included in the model, the simulations generate an absolute time base enabling the results to be compared to experimental data. An important advantage to this method is that non-reactive marker species can be used throughout the reaction scheme as indicated in **Supplementary Note 1**, and their accumulation as well as the occurrences of specific reaction and diffusion steps can be tracked as a function of time. These data can be differentiated to extract reaction and diffusion rates, providing in-depth views to how a reacting system evolves over time. The markers used in this work are shown in **Table S1**. The simulation code has an external stimulus function that enables selected reaction steps and diffusion steps to be modulated in time. This feature is used in this work to modulate reactant diffusion into the compartment system.

In this work, simulations were performed for a total reaction time of 20 s for the simple channel model, and 10s for the connected compartments model. The simulation outputs are a full set of concentrations as a function of space and time throughout the entire system, as well as catalytic current densities as a function of position and markers and occurrences information on the various reaction and diffusion steps taking place. Simulation results are exported for analysis using a spreadsheet to calculate rates, and for visualization using ParaView.[15]

Supplementary Note 5: Comparative timescales of intramolecular electron transfer (ET) vs substrate turnover in *Desulfovibrio vulgaris* formate dehydrogenase

Metal enzymes of the formate dehydrogenase (FdhAB) family are comprised of Fe-S clusters that act as initial points of reduction/oxidation and convey charge to/from a molybdenum, tungsten or NADPH-dependent catalytic site that drives the reversible conversion of CO_2 and formate. We consider here how ET and the chemical processes are coupled in *Desulfovibrio vulgaris*, where the metal is tungsten.

Electrochemical redox mediators have been used to investigate the energetics of the overall conversion reactions:
E^0, methyl viologen $MV/MV^{+\cdot}$: -446 mV vs RHE[16]
E^0, benzyl viologen, $BV/BV^{+\cdot}$: -0.359 mV vs RHE[16]

Oliveira et al. report turnover rates for FdhAB formate oxidation (1310 s^{-1} at pH 7.6) using BV and CO_2 reduction to formate (315 s^{-1} at pH 6.9) using MV.[17]

Nernstian adjustment of the redox values of these mediators for the assay pH used by Oliveira et al yields:

E, methyl viologen $MV/MV^{+\cdot}$: -440 mV at pH 6.9
E, benzyl viologen, $BV/BV^{+\cdot}$: -0.394 mV at pH 7.6

Driving forces (free energies ΔG^0 at E_0) for formate oxidation and CO_2 reduction to formate are then calculated according to $E_{rxn} = E_{red} - E_{ox}$:

Formate oxidation (pH 6.9): -440 mV – (-420 mV) = -20 mV driving force (ΔG^0)
CO_2 reduction (pH 7.6): -420 mV – (-394 mV) = -26 mV driving force (ΔG^0)

Estimation of intramolecular electron transfer rates can then be determined using the semiclassical Marcus equation:[18]

$$k_{et} = \frac{2\pi}{\hbar\sqrt{4\pi\lambda k_B T}} |H_{AB}| e^{\frac{-(\Delta G^0 + \lambda)^2}{4\lambda k_B T}}$$
(4)

$$H_{AB} = |H_{AB}^0 e^{(-0.5\beta r - r0)}|^2$$
(5)

where λ is the reorganization energy, H_{AB} is the electronic coupling constant between species A and B, β is an attenuation coefficient, r is the distance between A and B, and r_0 is the distance of closest approach between them.

The preexponential factor in equation (4) comprises a unimolecular ET rate term (k_0) that generally assumes values on the order of 10^{13} s^{-1} where $r = r_0$. For all r, this simplification can be generalized to yield:

$$k_{et} = k_0 e^{(-\beta r - r0)} e^{\frac{-(\Delta G^0 + \lambda)^2}{4\lambda k_B T}}$$
(6)

Furthermore, reorganization energies for intramolecular protein transfers generally span the range λ = 0.3 – 1.0 eV. Using the driving force values above, we can calculate the range of ET rates likely assumed by intramolecular charge tunneling in *D. vulgaris* FdhAB (β = 1.4 Å^{-1}, r_0 = 3 Å, T = 293 K). If we assume single-step electron transfer between the surface Fe-S cluster in the electron transport relay, and the W active site, the distance is taken as r = 31 Å. The resulting values are presented in **Table S2**.

Table S2. Rate coefficients calculated using Eq (5) as a function of reorganization energy

Reaction	ΔG [eV]	λ = 0.3 eV (lower bound)	λ = 1.0 eV (upper bound)
$CO_2 + 2\ H^+ + 2\ e^- \rightarrow$ HCOOH	-0.026	$4x10^{-5}$ s^{-1}	$5x10^{-6}$ s^{-1}
HCOOH → CO_2 + 2 H_2	-0.020	$4x10^{-5}$ s^{-1}	$5x10^{-6}$ s^{-1}

These single-step electron transfer values are far too small to allow for observed rates of substrate turnover in *D. vulgaris* FdhAB which spans $10^2 – 10^3$ s^{-1}. As a result, it is necessary to decompose ET into discrete hops between the Fe-S clusters comprising the relay between the protein surface and the buried W active site. Data on the redox potentials of the individual clusters are unavailable for this protein. However, the rate of electron self-exchange at equilibrium ($\Delta G = 0$) provides a useful lower bound value on possible ET in this transport chain because of the absence of a driving force. Exploring the case of self-exchange (over the set range of likely reorganization energies 0.3-1.0 eV), the factor controlling rates between these clusters and the W active site is the site-site distance, r. The crystal structure of this protein (PDBID: 6SDR) reveals four through-space ET jumps between the terminal Fe-S cluster and the W-molydopterin active site, of lengths 10.1, 9.2, 10.4 and 6.3 Å, as shown in **Figure S3**.

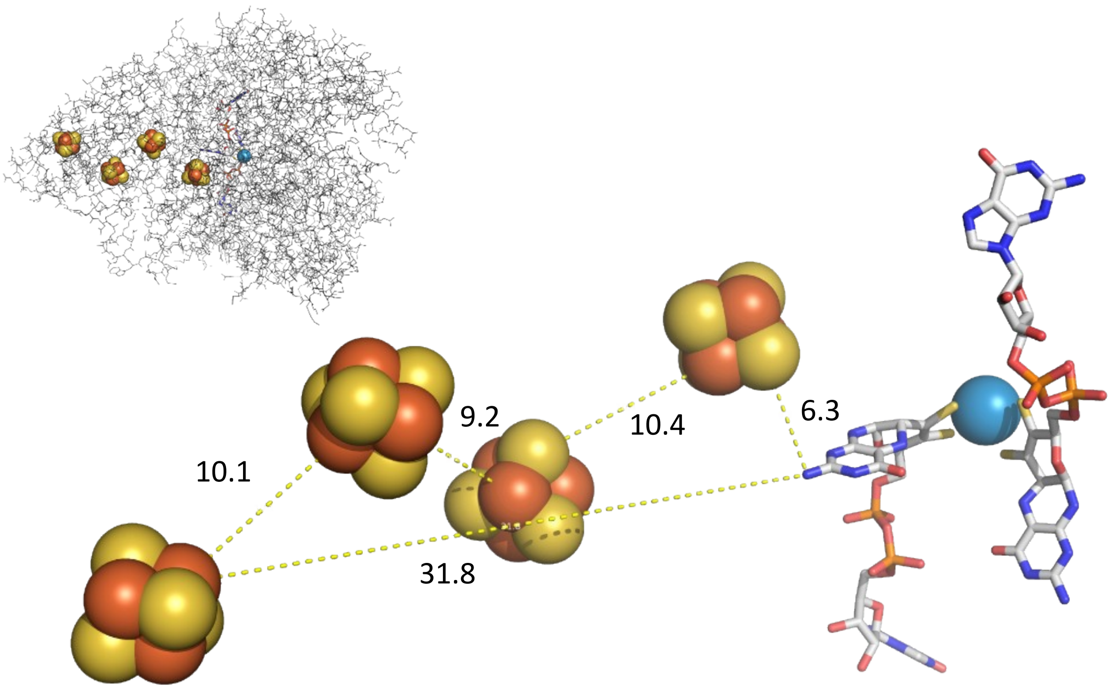

Figure S3. The structure of the electron transfer relay of *Desulfovibrio vulgaris* formate dehydrogenase (PDBID 6SDR). Charge propagation from the terminal Fe-S cluster located at the protein surface (far left) to the W active site (blue sphere) proceeds in discrete electron-hopping steps, with distances noted in the figure. Single step tunneling from the terminal Fe-S cluster to the active site edge is 31.8 Å, a distance that yields ET rates that are too slow to account for observed redox kinetics of the enzyme.

ET jump rates estimated for each step in the through-space route shown in **Figure S3** using eq (6) with the above values for r and parameter values of $\beta = 1.4$ Å^{-1}, $r_0 = 3$ Å, T = 293 K are presented in **Table S4**. Rates are the same for back and forward (formate oxidation and CO_2 reduction) reactions as a result of the equilibrium self-exchange condition (zero driving force).

Table S4. Estimated self-exchange rates for ET hopping steps.

ET Jump	ΔG [eV]	λ = 0.3 eV (lower bound)	λ = 1.0 eV (upper bound)
$Fe-S_1 \rightarrow Fe-S_2$ (10.1 Å)	0	$1.6x10^8$ s^{-1}	$1.8x10^7$ s^{-1}
$Fe-S_2 \rightarrow Fe-S_3$ (9.2 Å)	0	$6.6x10^8$ s^{-1}	$7.2x10^7$ s^{-1}
$Fe-S_3 \rightarrow Fe-S_4$ (10.4 Å)	0	$1.2x10^8$ s^{-1}	$1.4x10^7$ s^{-1}
$Fe-S_4 \rightarrow$ W-MGD (6.3 Å)	0	$3.8x10^{10}$ s^{-1}	$4.2x10^9$ s^{-1}
Overall: $Fe-S_1 \rightarrow$ W-MGD	0	$6x10^7$ s^{-1}	$7x10^6$ s^{-1}

These results point to overall electron hopping rate for the entire path that ranges between k_{et} = $6x10^7$ s^{-1} and k_{et} = $7x10^6$ s^{-1} for $0.3 < \lambda < 1.0$ eV. This limiting-rate of electron-self exchange in the FdhAB relay, using reorganization energies typical of ET proteins, yields ET rates that can accommodate the observed 2-electron substrate turnover rates by FdhAB at near-equilibrium driving forces achieved using viologen mediators (20-60 mV). In line with our assertion of the importance of timed charge and/or substrate delivery, which may be achieved by pulsing, comparison of these ET tunneling rates with those for actual substrate turnover suggest that tunneling steps in this enzyme occur on timescales of the order 10^4 to 10^5 times faster than the actual rate of substrate conversion (at near-equilibrium driving forces). Interestingly, this would suggest that electron transport in *D. vulgaris* FdhAB is a transient with respect to substrate conversion, with a pulse frequency of magnitudes ~10-100 kHz relative to the 2-electron substrate turnover.

References

1. F. A. Houle, J. Yano and J. W. Ager, Hurry Up and Wait: Managing the Inherent Mismatches in Time Scales in Natural and Artificial Photosynthetic Systems, *Acs Catal*, 2023, **13**, 7139-7158.
2. S. R. Li, S. Kwon, W. A. G. Goddard and F. A. Houle, A stochastic description of pH within nanoscopic water pools, *Cell Rep Phys Sci*, 2023, **4**, ARTN 101458.
3. K. L. Yang, R. Kas and W. A. Smith, In Situ Infrared Spectroscopy Reveals Persistent Alkalinity near Electrode Surfaces during CO Electroreduction, *J Am Chem Soc*, 2019, **141**, 15891-15900.
4. A. Arts, M. T. de Groot and J. van der Schaaf, Separating kinetics and mass transfer in formic acid and formate oxidation on boron doped diamond electrodes, *J Electroanal Chem*, 2020, **876**, ARTN 114721.
5. S. P. Cadogan, G. C. Maitland and J. P. M. Trusler, Diffusion Coefficients of CO2 and N2 in Water at Temperatures between 298.15 K and 423.15 K at Pressures up to 45 MPa, *J Chem Eng Data*, 2014, **59**, 519-525.
6. D. L. Wise and G. Houghton, Diffusion Coefficients of Neon Krypton Xenon Carbon Monoxide and Nitric Oxide in Water at 10-60 Degree C, *Chem Eng Sci*, 1968, **23**, 1211.
7. R. N. Massad, T. P. Cheshire, C. Q. Fan and F. A. Houle, Water oxidation by a dye-catalyst diad in natural sunlight: timing and coordination of excitations and reactions across timescales of picoseconds to hours, *Chem Sci*, 2023, **14**, 1997-2008.
8. L. D. Chen, M. Urushihara, K. R. Chan and J. K. Norskov, Electric Field Effects in Electrochemical CO2 Reduction, *Acs Catal*, 2016, **6**, 7133-7139.
9. W. D. Hinsberg and F. A. Houle, Kinetiscope, www.hinsberg.net/kinetiscope, (accessed Oct 1, 2024).

10. F. A. Houle, Reaction-Transport Coupling in a Nanostructured Porous Electrode, *J Phys Chem C*, 2019, **123**, 14459-14467.
11. F. A. Houle, Adaptive response by an electrolyte: resilience to electron losses in a dye-sensitized porous photoanode, *Chem Sci*, 2021, **12**, 6117-6128.
12. D. L. Bunker, B. Garrett, T. Kleindienst and G. S. Long, Discrete Simulation Methods in Combustion Kinetics, *Combust Flame*, 1974, **23**, 373-379.
13. D. T. Gillespie, Exact Stochastic Simulation of Coupled Chemical-Reactions, *J Phys Chem-Us*, 1977, **81**, 2340-2361.
14. *USA Pat.,* US Patent 5,826,065, 1998.
15. ParaView 5.12.0, https://www.paraview.org/, (accessed 2/25/2025).
16. L. Michaelis and E. S. Hill, The viologen indicators, *J Gen Physiol*, 1933, **16**, 859-873.
17. A. R. Oliveira, C. Mota, C. Mourato, R. M. Domingos, M. F. A. Santos, D. Gesto, B. Guigliarelli, T. Santos-Silva, M. J. Romao and I. A. C. Pereira, Toward the Mechanistic Understanding of Enzymatic CO2 Reduction, *Acs Catal*, 2020, **10**, 3844-3856.
18. R. A. Marcus and N. Sutin, Electron Transfers in Chemistry and Biology, *Biochim Biophys Acta*, 1985, **811**, 265-322.
